# Molecular Control of Follicular Helper T cell Development and Differentiation

**DOI:** 10.3389/fimmu.2018.02470

**Published:** 2018-10-25

**Authors:** Haijing Wu, Yaxiong Deng, Ming Zhao, Jianzhong Zhang, Min Zheng, Genghui Chen, Linfeng Li, Zhibiao He, Qianjin Lu

**Affiliations:** ^1^Hunan Key Laboratory of Medical Epigenomics, Department of Dermatology, Second Xiangya Hospital, Central South University, Changsha, China; ^2^Immunology Section, Lund University, Lund, Sweden; ^3^Department of Dermatology, Peking University People's Hospital, Beijing, China; ^4^Department of Dermatology, The Second Affiliated Hospital, Zhejiang University School of Medicine, Hangzhou, China; ^5^Beijing Wenfeng Tianji Pharmaceuticals Ltd., Beijing, China; ^6^Department of Dermatology, Beijing Friendship Hospital, Capital Medical University, Beijing, China; ^7^Department of Emergency, Second Xiangya Hospital of Central South University, Changsha, China

**Keywords:** Tfh, Bcl-6, Blimp-1, transcription factors, epigenetics

## Abstract

Follicular helper T cells (Tfh) are specialized helper T cells that are predominantly located in germinal centers and provide help to B cells. The development and differentiation of Tfh cells has been shown to be regulated by transcription factors, such as B-cell lymphoma 6 protein (Bcl-6), signal transducer and activator of transcription 3 (STAT3) and B lymphocyte-induced maturation protein-1 (Blimp-1). In addition, cytokines, including IL-21, have been found to be important for Tfh cell development. Moreover, several epigenetic modifications have also been reported to be involved in the determination of Tfh cell fate. The regulatory network is complicated, and the number of novel molecules demonstrated to control the fate of Tfh cells is increasing. Therefore, this review aims to summarize the current knowledge regarding the molecular regulation of Tfh cell development and differentiation at the protein level and at the epigenetic level to elucidate Tfh cell biology and provide potential targets for clinical interventions in the future.

## Introduction

**Figure 1:**
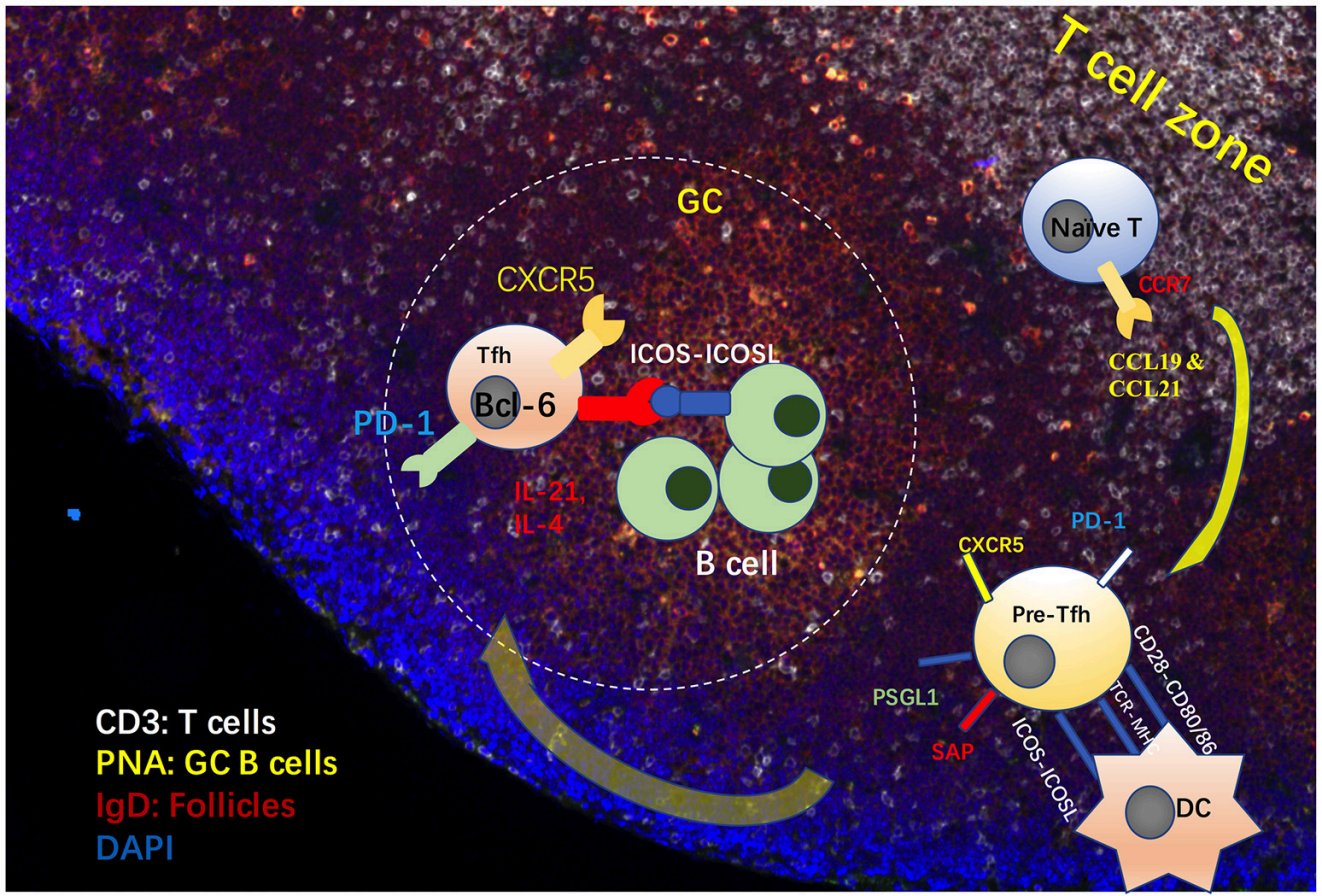
Process of Tfh cell differentiation and migration in GCs. Under the effects of CCL19 and CCL21, expression of the receptor CCR7 on naïve CD4^+^ T cells allows these cells to migrate into T cell zones in the secondary lymph nodes. With stimulation from antigens and CD80, CD86 and ICOSL expressed on dendritic cells (DCs), these cells differentiate into pre-Tfh cells, with high expression of CXCR5, PD-1 and signaling lymphocytic activation molecule adapter protein (SAP) and low expression of CCR7 and P selectin glycoprotein ligand 1 (PSGL1). Generally, Tfh cells provide signals for B cell maturation, differentiation and survival via ICOS, CD40L, IL-4, and IL-21 (16, 17). ICOS and ICOSL ligation is involved in Tfh-B cell interactions, which promotes calcium spikes in T-cells and CD40-CD40L signaling in B cells.

A subset of CD4^+^ T cells, which help B cells and are a resident in B follicles, has been described in the early 1990s (1–4). The existence of follicular helper T (Tfh) cells was proposed in 2000 (5, 6). However, the existence of these cells was not widely accepted until the identification of the Tfh cell linage-specific transcription factor, B-cell lymphoma 6 protein (Bcl-6), in follicular T cells in 2009 (7, 8). High expression of CXCR5 and low expression of CCR7 enable T cells to enter and stay in germinal centers (GCs) (6, 9–11). Bcl-6 deficient T cells have been shown to fail to differentiate into follicular helper T cells (8), indicating the importance of Bcl-6 in the determination of Tfh cell fate. Under the effects of CCL19 and CCL21, expression of the receptor CCR7 on naïve CD4^+^ T cells enables these cells to migrate into T cell zones in the secondary lymph nodes (9, 12). With stimulation from antigens and CD80, CD86 and ICOSL expressed on dendritic cells (DCs), these cells differentiate into pre-Tfh cells with high expression of PD-1, CXCR5 and signaling lymphocytic activation molecule adapter protein (SAP) (13) and low expression of CCR7 and P selectin glycoprotein ligand 1 (PSGL1) (14, 15) (Figure 1). Generally, Tfh cells provide signals for B cell maturation, differentiation and survival via ICOS, CD40L, IL-4, and IL-21 (16, 17). ICOS and ICOSL ligation is involved in T-B cell interactions, further promoting calcium spikes in T-cells and CD40-CD40L signaling in B cells (18). ICOS-deficient T cells fail to express CXCR5 and are unable to migrate into follicles, a finding also observed during antibody-blockade of ICOS-ligand (19, 20). PD-1 has been found to limit the number of Tfh cells (21). More evidence is needed to address the role of PD-1 in the migration and function of Tfh cells. SAP has been found to stabilize the interaction between B cells and Tfh cells (22). Therefore, Tfh cells can be distinguished from Th1, Th2 and Th17 cells using surface markers with a profile of CCR7^lo^PSGL1^lo^CXCR5^hi^PD-1^hi^ICOS^hi^. Activated by antigens and ICOSL expressed by DCs, the expression of Bcl-6 is upregulated in CD4^+^ T cells, and it represses other Th cell transcription factors, such as T-bet, GATA-3, and RORγT. Next, Bcl-6 promotes the transcription of Tfh cell migration and function-related genes, such as CXCR5, PD-1, and CXCR4 (8).

Tfh cells have been found to be regulated by a complex network of transcription factors, including the Bcl-6-Blimp1 axis, STAT1, STAT3, STAT4, STAT5b, B-cell activating transcription factor (Batf), v-maf avian musculoaponeurotic fibrosarcoma oncogene homolog (c-Maf), interferon regulatory factor 4 (IRF4), Achaete-scute homolog 2 (Acl2), and T-cell-specific transcription factor 1 (TCF-1)-lymphoid enhancer binding factor 1 (LEF-1). Since the study of Tfh cells began, certain proteins have been identified to participate in the development of Tfh cells. In addition, such cytokines as IL-2, IL-6, IL-7, IL-9, IL-12, IL-21, IL-23, IL-27, and TGF-β have been reported to enhance or impair the differentiation and survival of Tfh cells. Therefore, this review will comprehensively describe the current knowledge of Tfh cells, hoping to provide potential targets for Tfh cell-mediated autoimmune disease.

### Transcription factor network

#### Bcl-6-Blimp-1 axis

The discovery of Bcl-6 in Tfh cells is a hallmark for the identification of Tfh cells. The essential role of Bcl-6 has been confirmed in a mice study, indicating that CD4^+^ T cells deficient in Bcl-6 fail to differentiate into Tfh cells (8). Forced expression of Bcl-6 in CD4^+^ T cells promotes the expression of CXCR5, CXCR4, and PD-1(8). Bcl-6 can bind to the promoters of Th1 and Th17 cell transcriptional regulators T-bet and RORγT, thereby repressing the production of IFN-γ and IL-17 (8). The key role of Bcl-6 in Tfh cell fate determination has been further confirmed in subsequent studies (23, 24), and one of them reveals that Bcl-6 regulates Tfh cell early differentiation in an IL-21- and IL-6-independent manner (24). Conversely, Bcl-6 can bind to the promoters and enhancers of several migration-related genes, such as CCR7, CCR6, PSGL-1, CXCR5, CXCR4, PD-1, and SAP (24, 25). In addition, Bcl-6-targeted genes are enriched in the MAPK and JAK-STAT signaling pathways and cytokine-cytokine receptor ligations, which are involved in cell activation, metabolism and maintenance (26).

Blimp-1 has been found to be a critical antagonist for Tfh cell differentiation but an important transcription factor for other effector cells, such as Th1, Th2, Th17, and regulatory T cells (7). In a mouse study, Blimp-1-deficient CD4^+^ T cells preferentially develop into Tfh cells *in vivo*, while Blimp-1-expressing CD4^+^ T cells failed to aid in germinal center formation (7). Therefore, Tfh cell differentiation is believed to be a distinct pathway independently regulated by Bcl-6, in contrast to other effector T cells regulated by Blimp-1 (27). More importantly, constitutive expression of Blimp-1 has an inhibitory effect on Bcl-6 expression and thus represses Tfh cell differentiation (7), indicating that Bcl-6 and Blimp-1 are antagonistic regulators in Tfh cells. The results in B cells might shed light on this mechanism. In plasma cell differentiation, the release of Bcl-6-bound histone deacetylases (HDACs) may increase the histone acetylation levels on the promoter region of Blimp-1, promoting the expression of this gene (28, 29). Thus, HDACs might be the competitive substrate for these two genes. In autoimmune status, Bcl-6 deficiency in lupus-prone mice has been found to impair lupus-like symptoms (30), and increased Bcl-6 has been observed in lupus circulating Tfh-like cells, which is positively correlated with disease activity (31).

#### Bcl-6 and STAT5

Similar to the Bcl-6-Blimp-1 axis, Bcl-6 and STAT5 also inhibit each other due to their overlapping binding sites in many Tfh cell-related genes, including *Socs2, IL7r*, and *Tcf7*. In a mouse study, Bcl-6 has been found to repress both IL-7R and STAT5 expression, as well as inhibiting IL-2-induced STAT5 activation (32). This inhibitory effect on STAT5 by Bcl-6 is due to the abrogation of STAT5 phosphorylation (32). In contrast, signals through IL-2-CD25 activate STAT5 and inhibit Bcl-6 and CXCR5 via inducing Blimp-1 (20, 33, 34), and lack of IL-2R signaling leads to Bcl-6 expression (35). A high concentration of IL-2 has been found to inhibit Bcl-6 expression in polarized Th1 cells, in which the Bcl-6 DNA-binding domain is masked by the T-bet-Bcl-6 complex and normally shows low levels of Bcl-6 expression in response to limited IL-2 (36). However, in response to low IL-2, besides increased Bcl-6 and IL-6R, Th1 cells can also increase the expression of IL-7R, which can repress Tfh-related genes, including *cxcr5* and *bcl6* via IL-7-dependent STAT5 activation (37). In addition, Bcl-6 in Tfh cells has been observed to have a decreased level of 5-hydroxymethylcytosine (5hmC), which might explain the markedly high level of Bcl-6 in Tfh cells (32). Conversely, Bcl-6 deficiency results in increased STAT5 signaling and promotes the differentiation of non-Tfh effector T cells. The inhibitory effects of STAT5 have been found to be Blimp-1-independent. In addition, inhibition of IL-2 results in the reduction of Blimp-1 expression (38), indicating that IL-2, STAT5 and Blimp-1 collaboratively inhibit Tfh cell differentiation (39).

#### STAT3

IL-21 and IL-6/STAT3 are first described to be essential for Th17 cell differentiation (40). Next, STAT3 has found to be critical for Tfh cell differentiation. The evidence come from the fact that reduced IL-21 production is reported in mouse STAT3-deficient T cells, and only a STAT3 mutation, rather than *Il12RB1*, reduce the frequency of Tfh cells *in vivo* (41). Similarly, in CD4^+^ T cell-conditional STAT3 knockout mice, fewer CXCR5^+^ Tfh cells, as well as defective GCs and reduced IgG and IgM antibody production, have been observed after KLH immunization (42, 43). In another study, the gene expression of *Cxcr5* and *Icos* is shown to be downregulated in STAT3-deficient mice, while the expression of Blimp-1 is increased (44). More importantly, cluster analysis showed that STAT3-deficient Ly6C^lo^ PSGL-1^hi^ T cells in the T cell zone more closely resemble Th1 cells, with a high expression of IFN-induced genes (44). More direct evidence is that STAT3 can form a complex with Ikaros zinc finger transcription factor Aiolos to regulate Bcl-6 expression (45). In a human study, rather than in a mouse system, TGF-beta has been found to provide critical additional signals for STAT3 and STAT4 to initiate Tfh cell differentiation (46), emphasizing the important role of STAT3 in Tfh cell development. Unlike the critical role of IL-6 in early Tfh cell differentiation, STAT3 deficiency fails to recapitulate the impaired Tfh frequency. However, in this study, STAT1 activity has been found to be required for Bcl-6 induction and initiating Tfh cell differentiation (47). In addition, STAT3 can suppress type 1 IFN induced CD25 expression and can compete with STAT5 to bind to the Bcl6 locus (48). However, it might be difficult to distinguish whether the effects of STAT3 is intrinsic to the Tfh cell or a reflection of diminished capacity for other cell subset differentiation. The forced overexpression of STAT3 in T cell may provide an explanation to this issue, which is still lacking at this moment.

#### TCF-1 and LEF-1

TCF-1 and LEF-1 belong to the TCF-LEF subfamily and have been well-documented to be necessary for the maturation of double negative T cells to the double positive stage in thymus. In addition, TCF-1 has been reported to restrain mature T cell-mediated Th17 responses via suppressing IL-17 expression (49). TCF-1 and LEF-1 have been reported as critical transcription factors in Tfh cell differentiation by two independent studies published in the same year (50, 51). The loss of either TCF-1 or LEF-1 in mice leads to defects in Tfh cells, and the depletion of both TCF-1 and LEF-1 results in the impairment of Tfh cell differentiation and GC formation. In addition, the important role of LEF-1 has been emphasized by the observation that forced LEF-1 expression promotes the differentiation of Tfh cells (51). In another study, TCF-1 and LEF-1 are revealed to regulate the Bcl-6/Blimp-1 axis. TCF-1 has been identified as a positive regulator for Bcl-6 and it displays negative effects on Blimp-1 via directly binding to the Bcl-6 promoter to form a complex and regulatory region known as intron 3 of *Prdm1* (51). In addition, TCF-1 has been found to upregulate IL-6R expression and inhibit IL-2R expression (51), indicating that TCF-1 might be upstream of STAT3 and STAT5. The exact function of LEF-1 in Tfh cells remains unclear. However, evidence shows that LEF-1 synergistically works with TCF-1 to regulate Tfh cells, and TCF-1 can inhibit LEF-1 expression (51). Furthermore, TCF-1 and LEF-1 have been found to promote early Tfh cell differentiation by maintaining the expression of IL-6Rα and gp130 and enhancing ICOS and Bcl-6 expression (52).

#### Ascl2

Ascl2 is a basic helix-loop helix (bHLH) transcription factor that has been reported to initiate Tfh cell differentiation via upregulating CXCR5 but not Bcl-6 in T cells *in vitro* (53). In addition, *in vivo*, Ascl2 can promote T cell migration to the border of B cell follicles and can promote Tfh cell differentiation by inhibiting Th1 and Th17 signature genes and upregulating Tfh cell-related genes (53). In other studies, Ascl2 has been shown to be responsible for low CD25 expression on regulatory follicular T cells (Tfr) (54). Ascl2 displays the active chromatin marker trimethylated histone H3 lysine 4 (H3K4me3), which has not been observed in other T cell subsets. In contrast, other Tfh cell-related genes, such as *Bcl-6, Maf, Batf*, and *Irf4*, are associated with H3K4me3 in all T-cell subsets (55).

#### C-Maf

c-Maf, a member of the activator protein 1 (AP-1) transcription factor family, has been found to be highly expressed by Th17 and mature Tfh cells compared with CD4^+^ICOS^hi^CXCR5^−^ or CD4^+^ICOS^lo^CXCR5^−^ non-Tfh cells. During Th17 cell differentiation, IL-6 plus TGF-β or IL-21 plus TGF-β can increase the expression of c-Maf, which is ICOS-dependent (56). As mentioned before, Bcl-6 controls the expression of migration genes that are important for the migration of T cells to the follicles. However, the introduction of Bcl-6 cannot alter the production of IL-21 and IL-4, which are the key cytokines produced by Tfh cells. c-Maf has been found to affect the production of IL-21 and CXCR5 (57). In addition, c-Maf and Bcl-6 have been reported to cooperate in the expression of Tfh cell-related genes, such as CXCR4, PD-1, and ICOS (57). The selective loss of c-Maf expression in T cells leads to the inhibition of Tfh cell differentiation in response to vaccinations and bacteria, and it is also critical for high-affinity antibody secretion in vaccinated animals (58). In addition, in Tfh cells, c-Maf has been shown to positively regulate IL-4 production via binding to the conserved noncoding sequence 2 (CNS2) region of the IL-4 locus and via the induction of IRF4 (59–61); however, this effect is c-Maf-independent (61).

#### Batf

Batf is also a member of the AP-1 family, which lacks transcriptional activation domains (TADs). Batf has been found to be highly expressed by Tfh cells and directly regulates the expression Bcl-6 and c-Maf (62). The expression of Batf has been observed to be regulated by IL-4-STAT6 in Th9 cells and IL-6-STAT3 signaling in M1 mouse myeloid leukemia cells (63–65). In Batf-deficient mouse T cells, the expression of Bcl-6 and c-Maf decreased dramatically, and Bcl-6 alone is not sufficient for Tfh cell differentiation in the absence of Batf (62). In addition, Batf can cooperate with IRF4 along with STAT3 and STAT4 to promote IL-4 production in Tfh cells via binding to the CNS2 region in the IL-4 locus. BATF does not impair IL-4 in Th2 cells but only Tfh cells (61). However, other studies show that the loss of Batf impairs IL-4 production in both Tfh and Th2 cells (66, 67).

#### IRF4

IRF4 has been well-documented as an important transcription factor in the differentiation of helper T cells and B cells via promoting cell development (68). IRF4 expression in mouse T cells has also been found to promote GC formation by promoting Tfh cell differentiation (69). In IRF4 knockout mice, CD4^+^ T cells in lymph nodes and Peyer's patches failed to express Bcl-6 and Tfh cell-related genes. In addition, the adoptive transfer of wild-type Tfh cells cannot rescue the failed IRF4^−/−^ Tfh cell differentiation (69), indicating a critical role for IRF4 in Tfh cell development. In wild-type mice, IRF4 can interact with JUN and Batf to form a heterotrimer that can bind to AP1-IRF4 complexes and regulate Tfh cell differentiation (69). In another study, IRF4^−/−^ CD4^+^ T cells have impaired STAT3 binding and fail to differentiate into Tfh cells (70). In a recent study, the *Irf4* locus is reported to “sense” the intensity of TCR signaling to determine the *Irf4* expression level. The binding of IRF4 to divergent DNA sequences is regulated by the expression levels of IRF4 and controls Th cell fate determination (71). In Th2 cells, enhancers show a spectrum of occupancy by the Batf-IRF4 complex, which correlates with the sensitivity of gene expression to TCR signal strength (72). The adaptor molecule LAT has been revealed to export the repressor HDAC7 from the nucleus of CD4(+) T cells. The loss of LAT results in impaired TCR signal and the repression of HDAC7 targeted gene *Nur77* and *Irf4* (73). Furthermore, IRF4 has been reported to be induced in a TCR-affinity dependent manner, and it is critical for clonal expansion (74).

In addition, other transcription factors have also been reported to be involved in Tfh cell differentiation. Foxo1, which has been found to negatively regulate Tfh cell differentiation in the early stages of differentiation (75), has also been identified to positively promote Tfh cell differentiation in the late stage of this process (76). However, the molecular mechanism remains unclear. FOXP1 negatively regulates Tfh cell differentiation by directly inhibiting ICOS expression and IL-21 production (77). Kruppel-like factor 2 (KLF2), a transcription factor, has been found to be involved in T cell trafficking, survival and homeostasis. KLF2 deficiency has been linked with increased number of Tfh cells, and forced expression of KLF2 results in reduced Tfh cell differentiation and GC formation (78). KLF2 can negatively control Tfh cell differentiation by inhibiting the homing receptors, such as CXCR5, CCR7, S1PR1 and PSGL1 (79), via induction of negative regulators for Tfh cells, including Blimp-1, T-bet and GATA3 (78).

#### Other proteins regulating Tfh cell differentiation E3 ubiquitin ligase

Roquin is an RNA binding protein, which has been revealed to play a critical role in innate and adoptive immune systems. The lack of Roquin activity results in numerous autoimmune diseases, such as lupus and inflammatory bowel disease. It is well-known that *sanroque* mice, which have the mutant ROQUIN^M199R^ that promotes Tfh cells, show a spontaneous germinal center (GC) and accumulation of plasma cells (30, 80, 81). The ubiquitin E3 ligase Roquin-1 negatively regulates Tfh cell differentiation by recognizing and directly binding a cis-element in the 3' untranslated region of ICOS mRNA, thereby repressing ICOS expression (82). The combined loss of Roquin-1 and 2 results in spontaneous Tfh cell and germinal center development (83). Other Tfh-related genes, such as *Il6, Irf4, Ox40*, (84, 85) and *Ifng* (86), are repressed by Roquin. The loss of the RUNG domain of Roquin has been found to reduce the number of Tfh cells, which might be a result of impaired mTOR signaling (87) and reduced Bcl-6 expression (88). In addition, the E3 ubiquitin ligase Itch has also been reported to regulate Tfh cells by regulating the ubiquitination and degradation of Foxo1 (89), and the effect of Itch has been revealed to be upstream of Bcl-6, which is validated by the fact that forced Bcl-6 in Itch deficient mice can restore Tfh cell differentiation (89). Moreover, the E3 ubiquitin ligase Cullin3 acts as a negative regulator by directly binding to Bcl-6 and regulating the ubiquitination of histone proteins (90). Furthermore, in transplantation, herpesvirus entry mediator/B-and T-lymphocyte attenuator (HVEM/BTLA) signaling pathway has been found to be dispensable for the expansion of Tfh cells and formation of *de novo* host anti-donor isotype-specific antibodies (91).

#### Notch−1 and −2

The T cell-specific deletion of Notch-1 and Notch-2 results in the reduced number of fully mature Tfh cells and the absence of high-affinity Abs (92). These mature Tfh cells produce low levels of IL-21 and displayed low expression of Bcl-6 and C-Maf. However, the effect of the loss of Notch on Tfh cell differentiation is in an IL-4-independent manner (92). In a recent study, Notch signaling has been identified as an early lineage-determining factor between Tfh and Th2 cell fate (93). In addition, Delta-L 1/4-mediated signals to Tfh cells occur from stroma cells, and follicular dendritic cells are not required (93, 94). Fasnacht et al. (94) shows DLL4 in stromal cells is important for Tfh development. In a previous study, fibroblasts, rather than hematopoietic or endothelial cells, as niche cells, support Notch-2 driven differentiation of marginal-zone B cells, ESAMDCs, and Tfh cells (94).

### Surface molecule regulation

#### CXCR5

CXCR5 is a hallmark of Tfh cells that guides T cells to migrate to the B cell zone by binding to CXCL13 that is expressed by follicular dendritic cells (95). CXCL13 is expressed in the follicular mantle zone and not in the endothelial venules and paracortical T cell zone, where ligands for CCR7 exist. Unlike CCR7 ligands, CCL19 and CCL21, CXCL13 controls the segregation between T and B cells, rather than recruiting T cells and B cells to lymph nodes (96). Therefore, these CXCR5 ^hi^ T cells express a low level of CCR7, which helps these T cells to migrate to GCs (6, 9, 10, 97). Moreover, CXCR5 has been found to help the maintenance of PD-1 hi Tfh population in GCs (9). CXCR5-deficient mice have a low GC number and antibody production (95), which shows the important role of CXCR5 in Tfh cell differentiation. In addition to being controlled by Bcl-6, CXCR5 expression is also regulated by nuclear factor of activated T cells (NFAT2) (98).

#### ICOS/ICOSL

With signals from MHC-antigen-TCR and CD28 stimulation, ICOS expression is induced on activated T cells. Therefore, ICOS is not a reliable marker for Tfh cells not only due to its expression on precursor Tfh cells but also due to its high expression on activated T cells. Signals through ICOS-ICOSL are critical for Tfh cell differentiation, B cell survival and activation, antibody class switching and GC formation (99). In human Tfh cells, ICOS is used as a marker of GC Tfh cells (100). However, ICOS is probably not a reliable marker for GC Tfh cells in mice due to its similar expression in Tfh cells and precursor Tfh cells (101). It has been found that initial DC priming is sufficient to differentiate CXCR5^+^Bcl-6^+^ Tfh cells, but this process depends on consistent ICOS/ICOSL signaling from DCs (102). Further ICOSL signals from B cells are necessary for the complete differentiation and maintenance of GC-Tfh cells (14). ICOS has been found to be capable of regulating the migration of T cells to GCs via the induction of filopodia (17). Signals through ICOS/ICOSL activate phosphoinositide-3 kinase (PI3K) (103), which is also a critical kinase for Tfh cell differentiation via the AKT-mediated inactivation of FOXO (104). In addition, ICOS is able to maintain the Tfh cell phenotype via FOXO1-mediated KLF2 expression (79), and FOXO1 is also inhibited by ICOS-induced mTORc2 (75). ICOS signaling can also affect IL-21 production via c-Maf, thereby regulating Tfh cell differentiation (56). The importance of ICOS in Tfh cells has been demonstrated by a study showing that ICOS-deficient mice have impaired GCs, a reduced level of CXCR5^+^ memory T cells (19, 105), impaired immunoglobulin class switching and low levels of IL-4 when primed *in vivo* and restimulated *in vitro* with a specific antigen (106, 107).

#### OX40/OX40L

OX40 belongs to the TNFR family and is transiently expressed by T cells during chronic virus infection (108). OX40 has been found to play a critical role in Tfh cell differentiation. Reinforcing OX40 stimulation promotes the expression of Blimp-1 in LCMV-specific T cells and inhibits the differentiation of Tfh cells (108). However, OX40-deficient mice display impaired generation of Tfh cells and GCs (109), indicating that OX40 is important for the Tfh cell differentiation. Indeed, OX40L has been reported to contribute to lupus by promoting Tfh cell responses (110). In addition, TSLP-activated dendritic cells have been found to be able to induce Tfh cell generation via OX40L (111). In addition, OX40 can cooperate with ICOS to amplify Tfh cell development during vaccinia virus infection (112).

#### Other important surface markers

PD-1, which is usually expressed by exhausted T cells, is highly expressed on Tfh cells. PD-1/PD-Ls signals are generally considered as negative regulatory signals that dephosphorylate TCR signaling, thereby inhibiting activation and cytokine production by T cells (113). PD-1 expressed by Tfh cells is believed to balance the negative regulation from IL-2-mediated STAT5 signaling (114). In addition, Tfr cells also express PD-1, which regulates Tfr cells (115). In a recent report, PD-1 has been found to inhibit follicular T cell recruitment via limiting CXCR3 expression to confine Tfh cell localization in GCs and increase the stringency of GC affinity selection through PD-1-PD-L1 ligation (116). Cytotoxic T lymphocyte antigen 4 (CTLA-4) is another negative regulator of T cells that has been reported to be expressed by Tfh cells and Tfr cells (117). Tfr and Treg cells regulate Tfh cells via B7-1 and B7-2 binding to CTLA-4. Loss of CTLA-4 in Tfh cells results in the promotion of B cell responses (118). Moreover, mice deficient in the SLAM-associated protein (SAP) show impaired GC formation and defects in T-B cell interaction (22, 119–121). Although SAP deficient T cells can express Tfh markers initially, reduced Tfh cells have been found in GCs from SAP deficient mice (30, 122), suggesting that SAP is required for the generation of functional Tfh cells and the differentiation of Tfh cell contains multiple steps.

### Cytokine regulation

Signals from follicular DCs and the cytokine milieu produced by DCs provide instructions for Tfh cell differentiation. Various cytokines, including IL-6, IL-21, IL-12, IL-23, IL-2, TGF-β, IL-1β, can regulate Bcl-6, STAT5, and Blimp-1 expression via the JAK- STAT signaling pathway (55). In the early stage of human Tfh cell differentiation, IL-12, IL-23, and TGF-β initiate this process. In addition, other STAT3-activating cytokines, such as IL-1β and IL-6, support this process in the presence of IL-12, IL-23, and TGF-β. The precursors of Tfh cells share similarities with other Th subsets and can further differentiate into Th1 and Th17 cells dependent on the balance of cytokines (123). Following interactions with B cells, precursor cells can differentiate into Th1-like Tfh cells and Th17-like Tfh cells (123). In addition, some reports have shown that Tfh cells can express IFN-gamma and IL-4, which provides help for cytokine-driven patterns of immunoglobulin class switching (124). In some autoimmune conditions, such as an experimental autoimmune encephalomyelitis (EAE) model of multiple sclerosis (MS), cells that display a Tfh cell phenotype produce IL-17 (56), and during helminth infection, Tfh cells in lymph nodes produce IL-4 (124–126). These IL-4 producing Tfh cells located in B cell follicles are found to be functionally different form Th2 cells found in peripheral region (124). These IL-4 producing Tfh cells express a low level of GATA3 and no IL-13 (127).

It has been well-established that IL-6-mediated STAT3 activation is critical for IL-21 expression in TCR stimulated mice and human T cells (40, 128). STAT3 can also respond to IL-21 and IL-23. Following cytokine stimulation, STAT3 is phosphorylated by JAK and binds to the Bcl-6 promoter to further promote Bcl-6 transcription (129). In addition, IL-12 has been reported to induce Bcl-6 expression in human T cells via STAT4 activation and has a greater effect on IL-21 production compared to IL-6 and IL-21 (130). In addition, the IL-12-STAT4 pathway can also regulate CXCR5, ICOS, c-Maf, and Batf expression in human T cells (131, 132). TGF-β has been found to enhance the function of STAT3-STAT4 to help T cells to express CXCR5, ICOS, Bcl-6, c-Maf, IL-21, and Batf, as well as to repress the expression of Blimp-1 (42). However, in mice, TGF-β has been reported to have negative regulatory effects on Bcl-6 expression via mir-10a (133). The positive regulation of TGF-β might be restricted to human *in vitro* studies. However, in mice, cytokines and TCR stimulation are insufficient, and the T-B interaction is necessary to generate Tfh cells (134).

### Epigenetic regulations

Epigenetic regulation refers to a modification that will not change the DNA sequence but alters the gene expression through several modifications, such as DNA methylation, histone modification and non-coding RNA-mediated regulations. Increasing evidence has shown the cooperation between epigenetic modifications with transcription factors to determine T cell fate (135).

**Figure 2:**
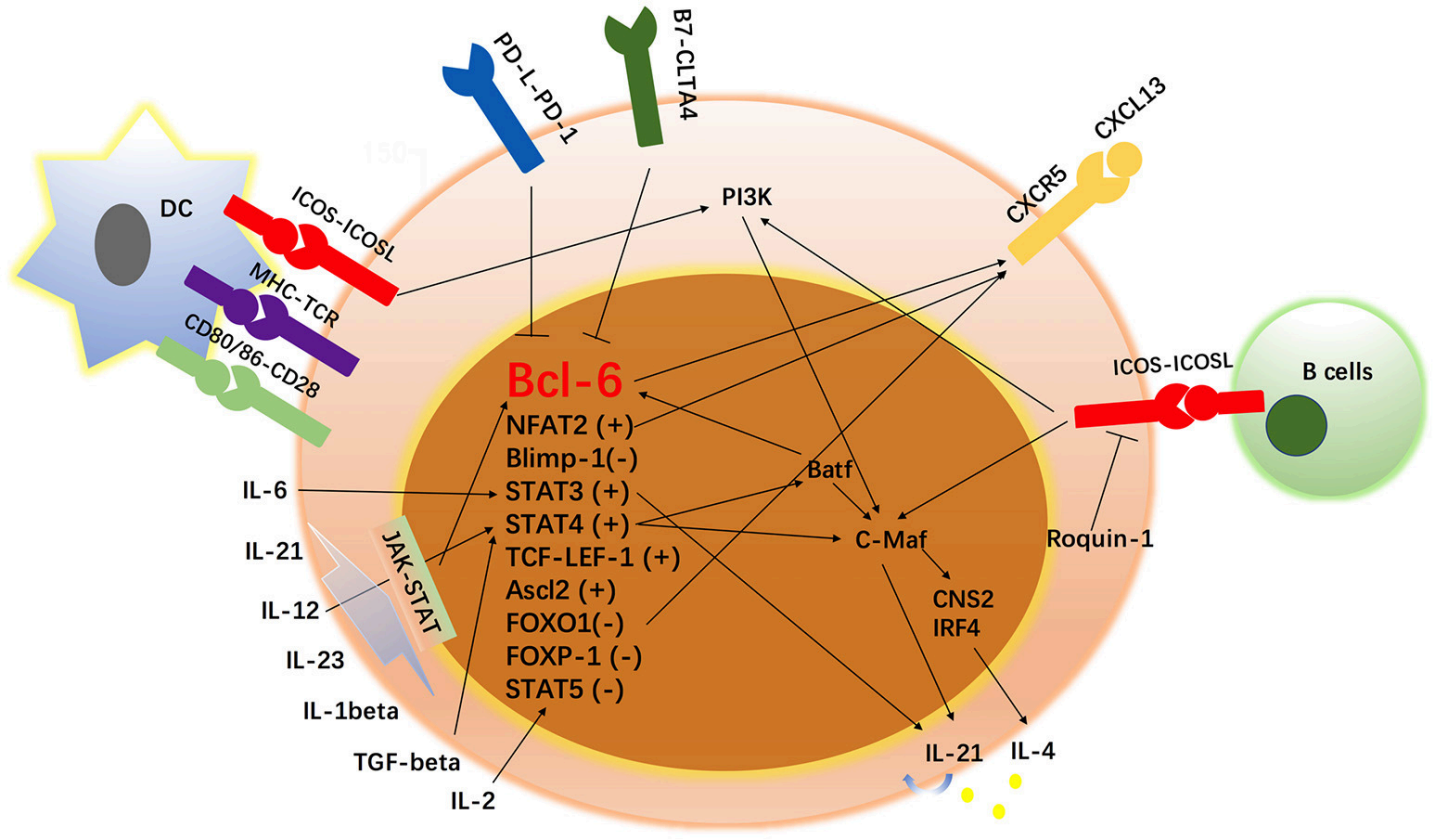
Network of transcription factors, cytokines and surface markers in Tfh cell regulation. In addition to the signals from surface markers, Tfh cells have been found to be regulated by a complex network of transcription factors, including the Bcl-6-Blimp1 axis, STAT1, STAT3, STAT4, STAT5, B-cell activating transcription factor (Batf), v-maf avian musculoaponeurotic fibrosarcoma oncogene homolog (c-Maf), interferon regulatory factor 4 (IRF4), Achaete-scute homolog 2 (Acl2), and T-cell-specific transcription factor 1 (TCF-1)-LEF-1, FOXO-1, FOXP-1, and NFAT-2. Since the study of Tfh cells began, some proteins have been identified to participate in the development of Tfh cells. In addition, cytokines such as IL-1 beta, IL-2, IL-6, IL-12, IL-21, IL-23, and TGF-β have been reported to be involved in the differentiation and survival of Tfh cells. “+” means positively regulates Tfh cell differentiation and “–” means negatively regulates Tfh cell development.

Unsurprisingly, Tfh cell differentiation is also regulated by epigenetic modifications. DNA methylation refers to silencing gene expression, and demethylation/hydroxymethylation is related to gene reactivation. In Tfh cells, Bcl-6 binding to gene loci has been found to be associated with reduced recruitment of translocation methylcytosine dioxygenase 1 (TET1), which is a hydroxymethyltransferase. Bcl-6 binding is also observed to result in reduced 5-hydroxymethylcytosine (5-hmC) (32), which is a mechanism for DNA demethylation. In addition, in our previous study, we found that IL-21 can increase TET2 enrichment on the promoter region of Bcl-6, which might explain the increased levels of Bcl-6 in lupus T cells (31). In addition, methylated H3K27 has been reported to prevent Tfh-related gene expression, while the H3K27me3 demethylase UTX sustains Tfh cells and antibody production (136). Positive histone modifications have been detected at the *Bcl-6* locus in Tfh cells, but negative marks are present at *Bcl-6* in other Th subsets (137). In addition, positive and negative histone modifications can be detected on *Prdm1* in all Tfh cell populations. These positive and negative histone modifications might provide clues for Tfh cell plasticity. miRNAs, which are non-coding RNAs, regulate gene expression at the posttranscriptional and posttranslational levels. miRNAs silence gene expression by targeting the 3'-untranslated regions of mRNA, causing mRNA cleavage and translational repression. In Tfh cells, the miR-17-92 cluster has been observed to be downregulated, which might contribute to the overexpression of Bcl-6 (138). miR-155 can regulate Tfh cell accumulation in miR-146a-deficient mice, resulting in abnormal Tfh cell accumulation (138). miR-146a can directly targets ICOS and the overexpression of ICOS mediated by the loss of miR-146a results in spontaneous and cell-autonomous Tfh cell accumulation (139). The molecules regulating Tfh cell differentiation are summarized in Figure 2.

Increasing evidence has shown the plasticity of Tfh cells, which can be explained by epigenetic regulations. Tfh cells display repressive histone markings (H2K27me3) on *Il4, Ifng*, and *Il17a*, while permissive active chromatin H3K4me3 on *Il21* locus (137, 140). Interestingly, the evidence that Tfh cells can produce effector T cell cytokines in response to the polarization cytokines and maintain the ability to produce IL-21 (137), can be explained by the fact that Tfh cells also display detectable H3K4me3 on *Tbx21, Gata3*, and *Rorc* locus (137). The positive H3K4me3 has been observed on the *Bcl6* gene in Tfh cells from an *in vivo* and *ex vivo* system. Other *in vitro* differentiated Th cells also show permissive markers on *Bcl6*, which enables these cells to acquire Tfh cell phenotypes and the capacity to produce IL-21 (137).

## Conclusions

Tfh cell differentiation is regulated by multiple transcription factors, receptors, cytokines, and epigenetic modifications. Unlike other Th cells, mouse Tfh cells are difficult to generate *in vitro* by cytokines and TCR stimulation, possibly reflecting a requirement for T-B cell interactions. ICOS/ICOSL signals might be an underlying explanation for the difficulty mentioned above. In addition, the cytokines driving differentiation in mouse and human systems are different; for example, TGF-β is a negative regulator in mice but a positive regulator in human Tfh cells. Tfh cells are heterogenic populations. Certain Th1, Th2, and Th17-like Tfh cells have been identified in GCs. In addition, Tfr cells have also been reported and regulate Tfh cell homeostasis. In addition, in certain inflammatory sites, such as synovium from rheumatoid arthritis patients, non-classic Tfh-like cells have been identified, which are CXCR5^low^ but have high expression levels of Bcl-6, PD-1, and IL-21. Single-cell mRNA sequencing should facilitate studies aiming at dissecting Tfh cell subset heterogeneity and distribution in tissues and blood. Our understanding of epigenetic regulation of Tfh cells is limited. Due to the development of new technologies, new molecules might be identified in the near future.

## Author contributions

HW wrote the manuscript. YD, MZ, JZ, MZ, LL, and GC edited the manuscript. ZH and QL revised the manuscript.

### Conflict of interest statement

The authors declare that the research was conducted in the absence of any commercial or financial relationships that could be construed as a potential conflict of interest.
